# Role of Aortic Geometry on Stroke Propensity based on Simulations of Patient-Specific Models

**DOI:** 10.1038/s41598-017-06681-3

**Published:** 2017-08-01

**Authors:** Hyo Won Choi, Tong Luo, Jose A. Navia, Ghassan S. Kassab

**Affiliations:** 1 The California Medical Innovations Institute, San Diego, California United States of America; 2 Department of Surgery, Austral University, Buenos Aires, Argentina

## Abstract

Stroke is a life threatening event that is expected to more than double over the next 40 years. Atrial fibrillation (AF) has been reported as a strong independent risk factor for stroke. We have previously shown that a hemodynamic perturbation by AF or reduced cardiac output and cycle length may have a significant impact on clot trajectory and thus embolic stroke propensity through the left common carotid artery using an idealized aortic arch model. Here, we show the dependence of flow patterns and hence stroke propensity on geometry of patient-specific aortas. We performed computational fluid dynamics (CFD) simulations to determine the variations of AF-induced stroke propensity over various image-based patient-dependent aorta models. The results demonstrated that curvature pattern of aorta can play a determinant role in AF-induced stroke propensity alteration. Specifically, it was shown that the hemodynamic perturbation by AF considered led to substantial increase in stroke propensity (i.e., 2.5~3.8 fold elevation) for lower curvature angle <90° while the changes in stroke propensity by AF are negligible for higher curvature angle >90°. The present simulations suggest that aortic arch curvature is an important risk factor for embolic stroke which should be tested in future clinical trials.

## Introduction

Stroke is a medical emergency that occurs when a blood vessel in the brain bursts (i.e., hemorrhagic stroke), or more commonly, when a blockage develops (i.e., ischemic stroke) from embolization by clots or debris. Without oxygen, cells in the brain quickly die and the outcome can be serious disability or death. It has been reported that stroke incidence and costs are expected to rise substantially with the aging population^1^. It has also been reported that majority of strokes are ischemic stroke (i.e., >87% of all strokes)^2^ and cerebral embolism is the primary cause of ischemic stroke (i.e., 40–80%)^3, 4^.

Among various medical and life style risk factors for stroke, atrial fibrillation (AF) has been reported as a strong independent risk factor^5–12^. AF is the most common dysfunction in heart rhythm characterized as arbitrary irregular heartbeats. Indeed, it has been shown that AF may lead to a broad spectrum of abnormal cardiac hemodynamic parameters^13–16^. We have recently shown that various aspects of AF hemodynamics (e.g., reduced cardiac output and cycle length) affect the trajectory of a cardiogenic blood clot transported into the carotid artery and embolic propensity in an idealized aortic arch model^17^.

Flow patterns in aortic arch are generally complex and multidirectional and may be affected by the geometrical configuration of the aorta arch that has a wide array of anatomical variations. This may also significantly affect the clot motion and ultimately propensity of embolic events. Here, we carried out computational fluid dynamics (CFD) simulations to examine how hemodynamic perturbations by AF may vary in patient-dependent aorta models. The arch morphological variations in patient-specific aortic models were characterized by curvature and torsion to examine how these morphological features are related to AF-induced stroke propensity. To our best knowledge, this is the first study to examine the relationship between aorta morphology and corresponding propensity of stroke incidence using patient-specific aortic models. The present findings provide a working hypothesis for future prospective studies that consider aortic curvature as an important risk factor for stroke propensity.

## Results

### Effect of Clot Forces on Trajectory

**Figure 1:**
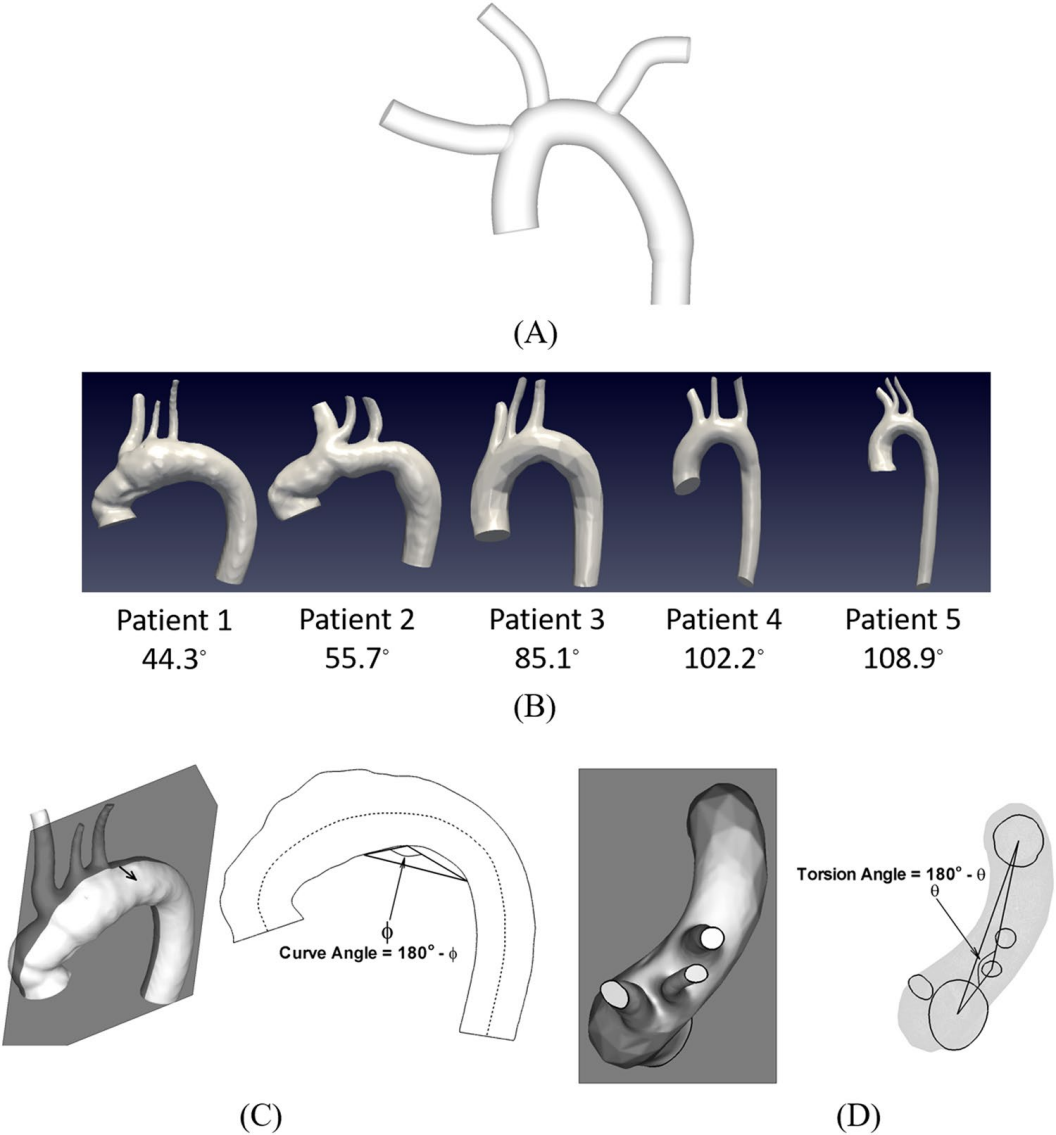
(**A**) Idealized aortic arch model. (**B**) Aorta models reconstructed from computed tomography (CT) scan images. The label indicates a curvature angle in each patient which is defined as sum of curve and torsion angles and indicative of global 3D curvature of aorta. (**C**) Curve angle of aorta or 180° − ϕ is defined as an angle made at the peak position of the inner aortic curve as projected onto the rough mid-plane passing through two centroid points of ascending aorta inlet and descending aorta outlet based on the previous study^35^. The black arrow in the left panel denotes a normal vector perpendicular to the mid-plane. Dashed line indicates a rough center line of aorta. (**D**) Torsion angle or 180° − θ which is defined as an angle at the left common carotid artery that is made by connecting three centroid points of ascending aorta, left common carotid artery, and descending aorta as projected in the descending aorta direction.

**Figure 2:**
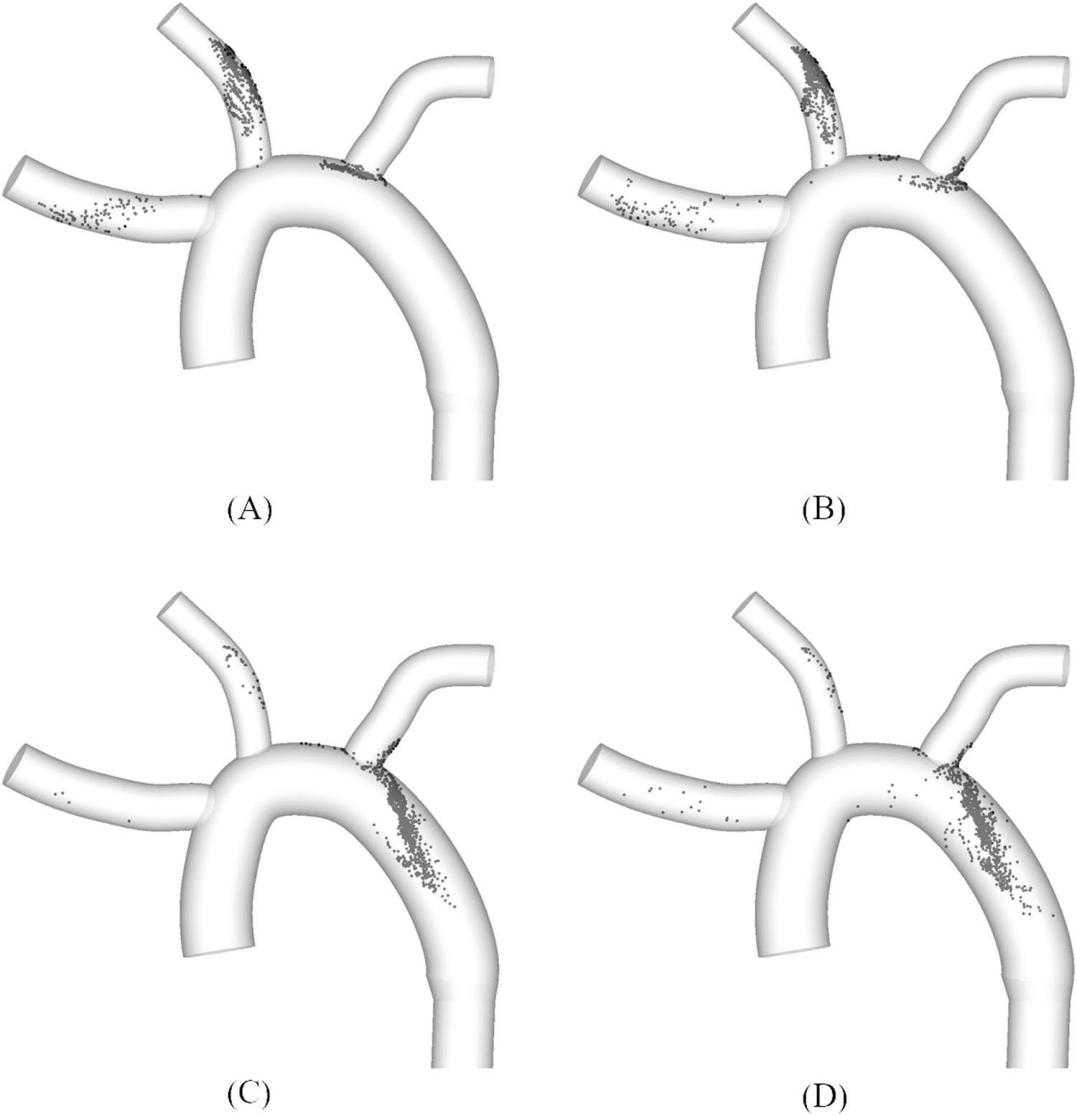
Distribution of clots within the idealized aorta model at the end of second systolic stage of a normal cardiac cycle after release of clots at the aortic inlet with application of (**A**) drag force + buoyancy force (**B**) drag force + buoyancy force + virtual mass force (**C**) drag force + buoyancy force + pressure gradient force, and (**D**) drag force + buoyancy force + virtual mass force + pressure gradient force on the clot.

The effect of forces exerted on a clot was systematically investigated using an idealized aorta model (Fig. 1A). Figure 2A through D represent the clot positions in the idealized aorta model at the end of second systolic stage of a normal cardiac cycle with drag and buoyancy force (Fig. 2A), drag, buoyancy and virtual mass force (Fig. 2B), drag, buoyancy and pressure gradient force (Fig. 2C), and drag, buoyancy, virtual mass and pressure gradient force (Fig. 2D). The results show that the number of clots transported to the left common carotid artery (LCCA) was significantly affected by how the forces applied on the clot are modeled. Among the various forces (i.e., drag, buoyancy, virtual mass, and pressure gradient), the simulations show that the pressure gradient force plays a dominant role in the clot trajectory under a normal cardiac hemodynamic condition.

### Patient-Dependent Stroke Propensity

**Figure 3:**
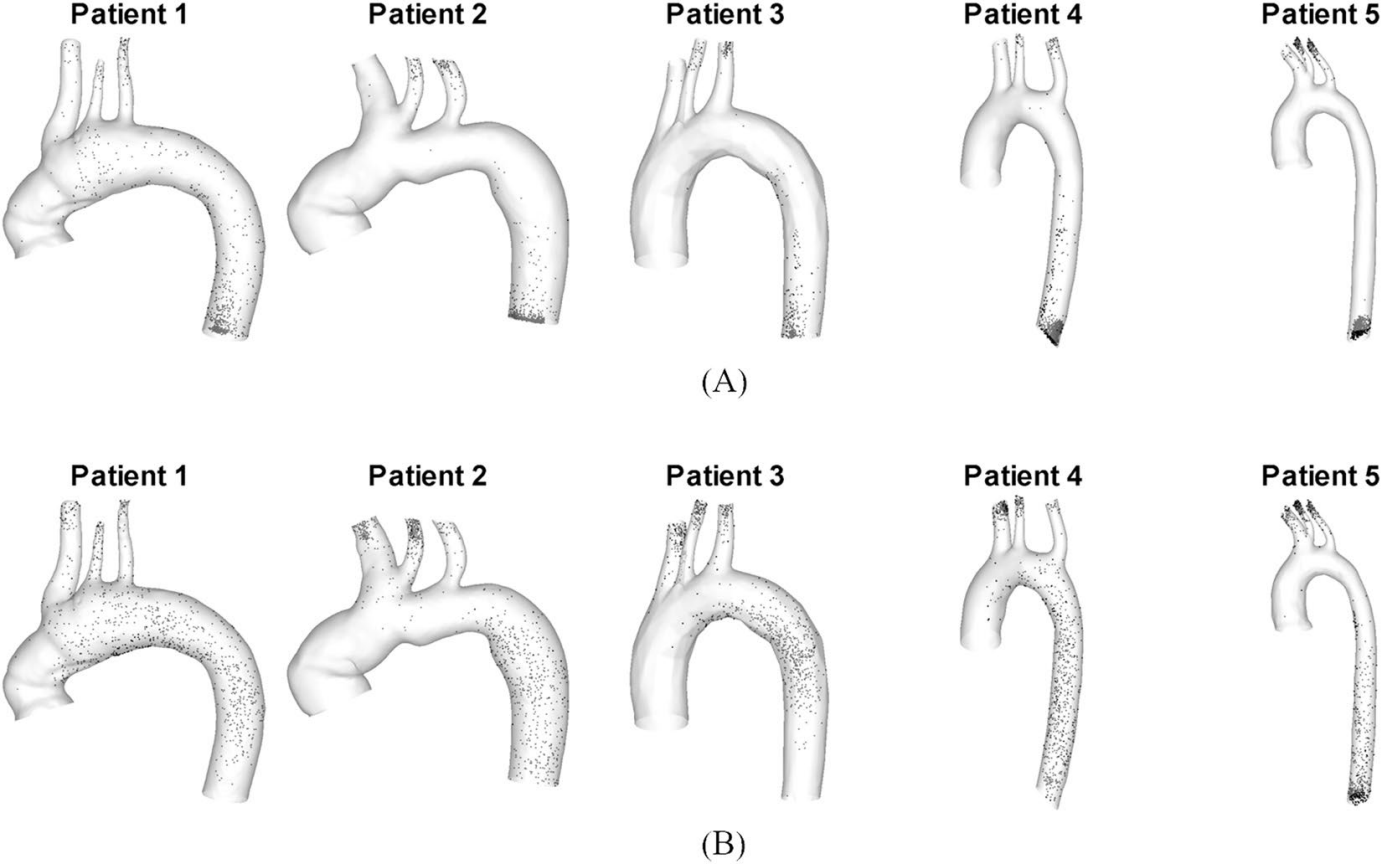
Distribution of clots at the peak stage of second cardiac cycle after released at the aortic inlet subject to the (**A**) normal and (**B**) AF flow condition within the aorta model reconstructed from patient 1 to 5.

The forces exerted on the clot including the force due to the pressure gradient in the blood flow field may vary significantly depending on the geometrical configurations of aortic model considered. Figure 3 indicates the clot positions at the end of second systolic stage of a cardiac cycle under normal and AF flow condition over five patient-specific aorta models shown in Fig. 1B. The results show a wide range of clot distributions over various geometrical configurations of the patient-dependent aorta models.

**Figure 4:**
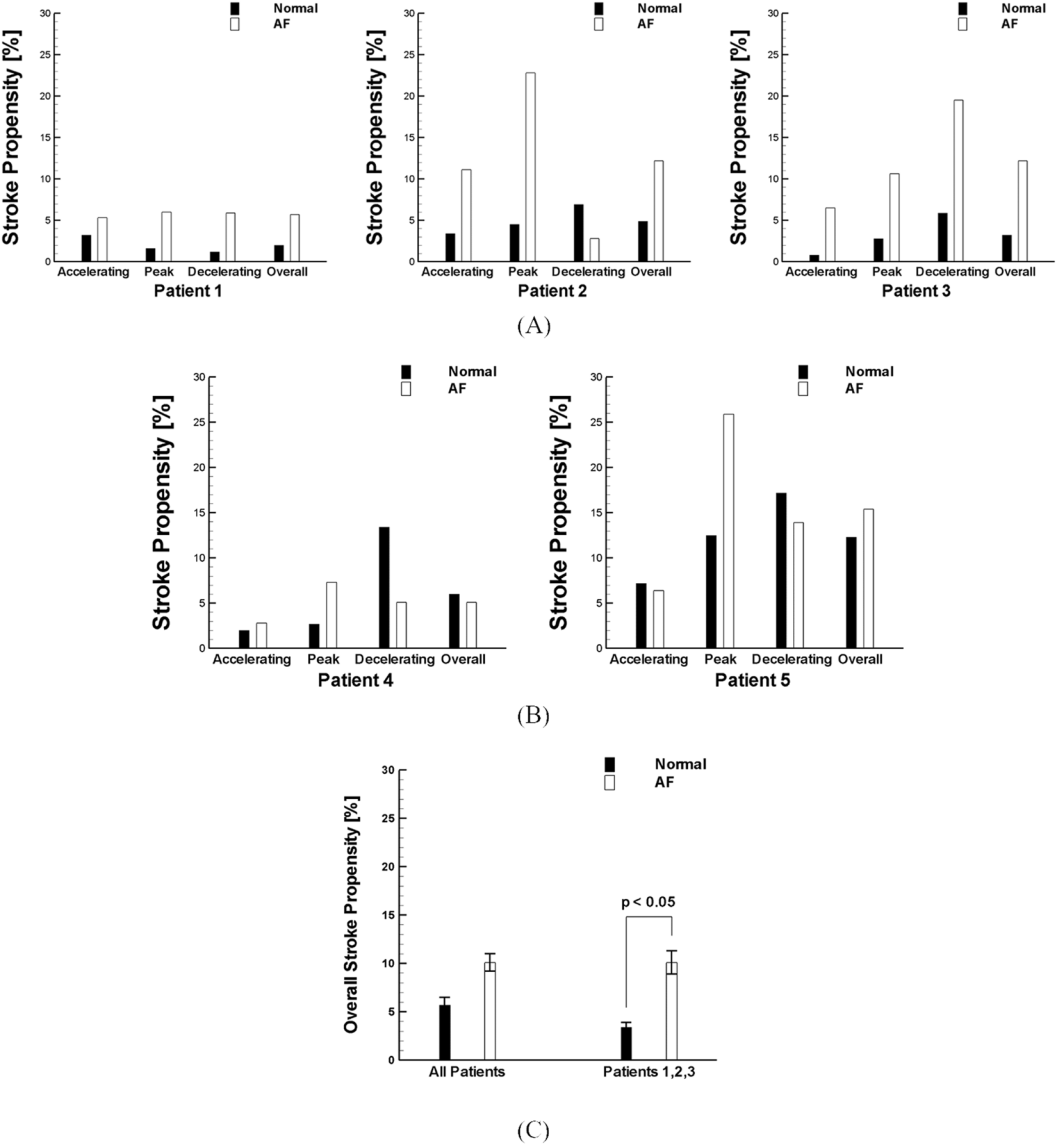
Stroke propensity through LCCA for various ejection instances (i.e., accelerating, peak, and decelerating stage of a cardiac cycle) and overall stroke propensity for various aorta models from (**A**) patient 1, 2, and 3 and (**B**) patient 4 and 5.

For quantitative comparison, the number of clots transported into the LCCA was calculated as an indicator of the stroke propensity for the five patients (Fig. 4). The results show that stroke propensity is a function of clot ejection dynamics as well as cardiac hemodynamic conditions and aortic structure. The simulation results show that stroke propensity is higher for the AF than normal condition when the clots are released at the peak stage of a cardiac cycle regardless of the patient-dependent aortic models. Although the results demonstrate that there is no significant difference in overall stroke propensity between normal and AF conditions for Patients 4 and 5 (Fig. 4B), the differences in overall stroke propensity between the AF and normal condition are evident for Patients 1, 2 and 3 (Fig. 4A). The statistical analysis indicates that the difference in overall stroke propensity between normal and AF condition is significant (p < 0.05, one-way ANOVA) for Patients 1, 2, and 3 while the difference is not statistically significant when all Patients including Patients 4 and 5 are considered (Fig. 4C). Also, the overall stroke propensity was shown to be higher for Patients 4 and 5 than Patients 1, 2, and 3 under normal conditions while no such correlation was observed under AF condition.

### Effect of Aortic Curvature on Stroke Propensity

**Figure 5:**
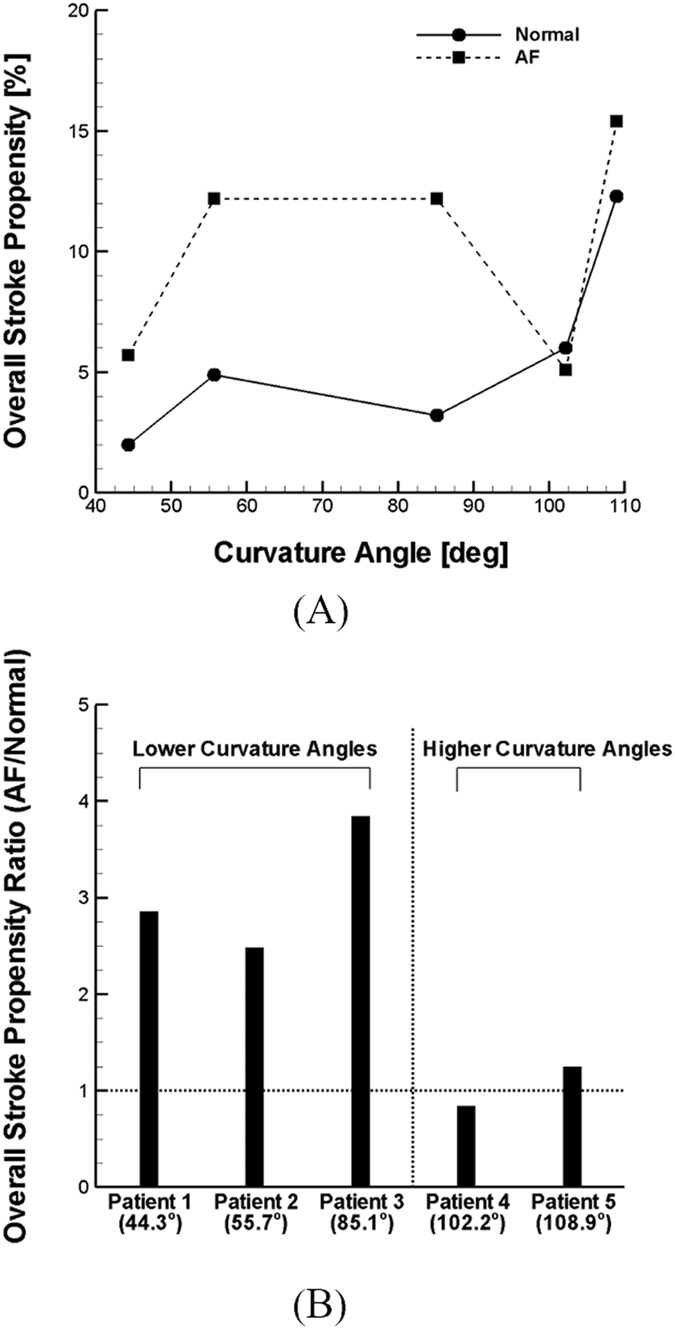
(**A**) Stroke propensity with curvature angle under the normal and AF flow condition. (**B**) Stroke propensity alteration induced by AF with curvature angle compared to the normal flow condition.

As illustrated in Fig. 5, the overall stroke propensity was plotted with the curvature angle of aortic arch defined as in Fig. 1C and D. The results suggest that the stroke propensity generally increases with the curvature angle under normal flow conditions while the relation between stroke propensity and the curvature angle was shown to be arbitrary under AF condition (Fig. 5A). Despite the non-monotonic relationship between the stroke propensity and curvature angle, the increase in stroke propensity under the AF condition was shown to be significant for lower curvature angles (Fig. 5B). Specifically, the ratio of stroke propensity under the AF condition relative to the normal condition was shown to range from 2.5–3.8 for the curvature angle <90° while it falls to near unity (i.e., 0.8–1.25) for the curvature angle >90°.

**Figure 6:**
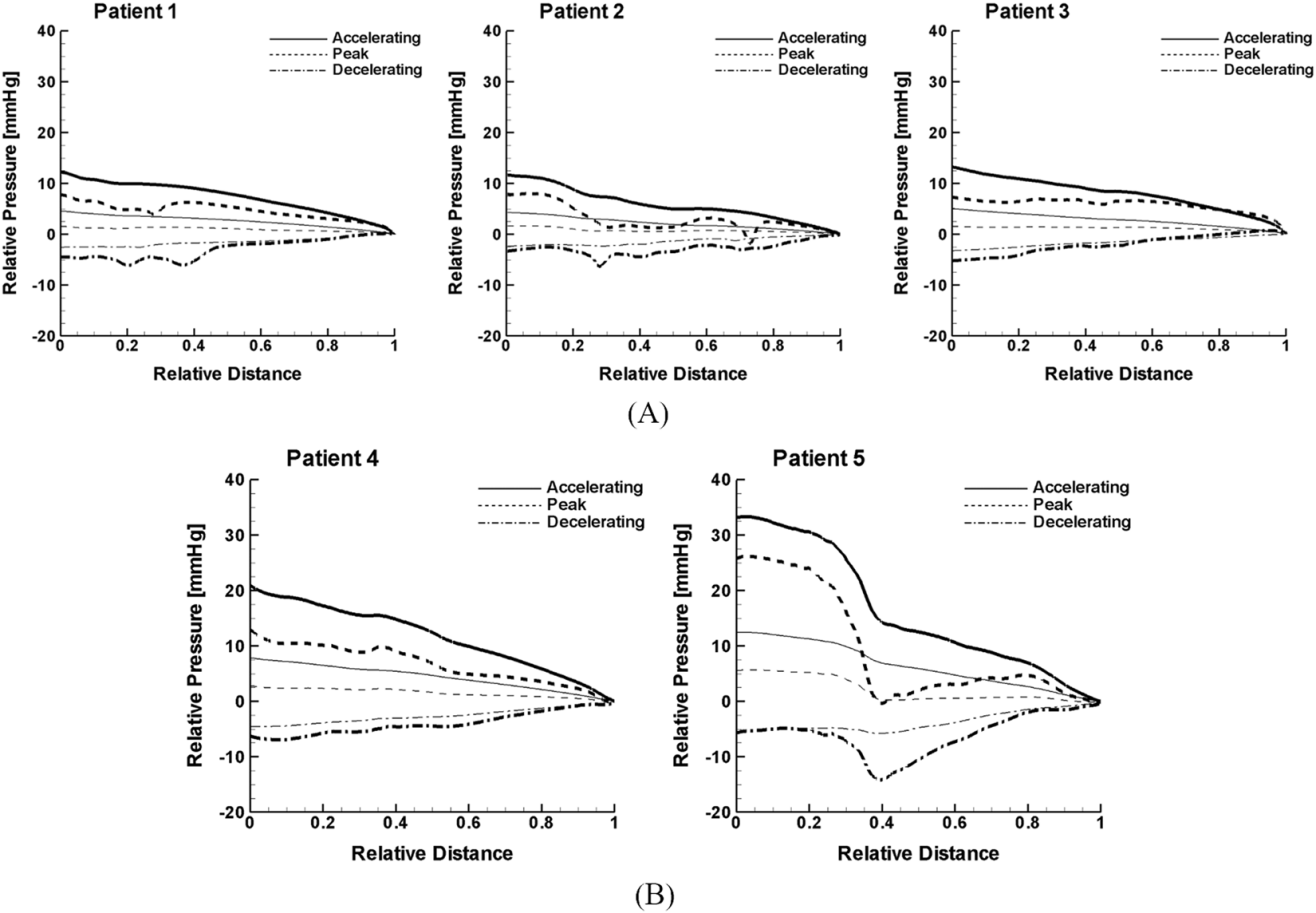
Pressure distribution with a cardiac cycle from accelerating or 0.25T_sys_ (solid lines) to peak or 0.5T_sys_ (dashed lines) and decelerating or 0.75T_sys_ (dashed dot lines) stage along the aorta center line relative to the descending aorta outlet as shown in Fig. 1C under the normal and AF flow condition in (**A**) patient 1, 2, and 3 and (**B**) patient 4 and 5. Thick and thin line denote the normal and AF flow condition, respectively. T_sys_ denotes the systolic period of a cardiac cycle.

The curvature angle of aorta configuration was shown to significantly affect the pressure field. Figure 6 represents the pressure distribution with a cardiac cycle from accelerating to peak and decelerating stage along the aortic curve relative to the descending aorta outlet (Fig. 1C) under normal and AF flow condition over the patient-specific aortic models considered. The results demonstrate that pressure drop along the aorta is generally higher for Patients 4 and 5 with higher curvature angles (Fig. 6B) than Patients 1, 2 and 3 with lower curvature angles (Fig. 6A) under both normal and AF condition. Also, changes in pressure along the aortic curve were shown to be steeper for Patients 4 and 5 than for Patients 1 to 3 under both normal and AF flow conditions. In addition, the results showed that the degree of pressure drop from the normal to AF condition is more dramatic for Patients 4 and 5 than for the Patients 1 to 3.

## Discussion

Although numerous patients suffer stroke not only in the US but also worldwide^18^ and various strategies including pharmacologic and implantable device approaches have been adopted for stroke prevention and treatment, little effort has been made to elucidate the role of hemodynamic forces in stroke incidence. Here, we show how aortic arch geometry, in patient-specific models, affects stroke propensity through the LCCA. A very interesting finding in the present study is that the overall aortic curvature is an important determinant of embolization to the LCCA albeit stroke propensity is likely regulated by a multitude of factors. Specifically, the present study suggests that the elevated risk of LCCA embolization induced by AF is critically dependent on the patient-specific geometry of aortic arch.

It has been previously demonstrated that the motion of a blood clot in blood flow is multifaceted such that the trajectory of a clot is governed by the interplay between hemodynamics and clot properties^17^. Despite such complexities, however, the simulations show that pressure gradient force can be a dominant factor that determines the trajectory of a clot and thus affects the stroke propensity (Fig. 2). Indeed, the trajectory of a clot was shown to be as diverse as the patient-dependent aortic configurations considered (Fig. 3).

Quantification of clot trajectories into LCCA indicated that stroke propensity is significantly affected by geometrical configurations of the aorta (Fig. 4). Despite the variations in stroke propensity pattern by clot ejection dynamics and hemodynamic conditions, the aorta structures considered were shown to be categorized by the overall stroke propensity due to AF-induced hemodynamic perturbation. In other words, elevated stroke propensity by AF was shown to be evident in some of the aortic configurations (Fig. 4A) which is consistent in our previous observation^17^ while such pattern was not observed in other aortic structures (Fig. 4B). This was statistically confirmed demonstrating that the AF-induced increase in overall stroke propensity is significant only for the aortic configurations with lower curvature angles (i.e., in Patients 1, 2, and 3; Fig. 4C). Among various geometrical aspects of aorta, the aortic curvature emerged as a key parameter for stroke propensity in AF.

Hence, a curvature angle was defined as an overall indicator of curvature and torsion of aortic arch (Fig. 1C and D). A nearly monotonic relationship between the stroke propensity and curvature angle was observed under normal conditions while no such correlation was found under the AF condition (Fig. 5A). The results, however, demonstrated that lower curvature angles (i.e., <90°) or relatively less tortuous configurations of aorta lead to significant increase in stroke propensity by AF while stroke propensity alteration by AF was shown to be negligible at higher curvature angles (Fig. 5B).

Both pressure drop and pressure gradient in the aorta were shown to generally increase with increased curvature angle for normal and AF condition. Elevated pressure gradient level in highly folded aorta configurations may affect clot trajectory and ultimately stroke propensity as suggested in Fig. 2. Indeed, stroke propensity was shown to increase with increased curvature angle (Fig. 5A) which is consistent with relation of pressure to curvature angle (Fig. 6) under normal flow condition. Although no consistent relationship was not found for AF flow condition, the results clearly demonstrate the impact of perturbed hemodynamics or pressure distribution by AF on clot trajectory directed into LCCA becomes prominent over aorta structures with relatively gradual curvature.

These findings suggest that the susceptibility of a patient-specific aortic structure to the risk of stroke in AF hemodynamic conditions is dependent on the flow patterns or spatial and temporal changes in pressure distribution in the aorta. Previous studies^19–23^ have reported progressive elongation, widening, and decreased curvature of aorta with aging (thoracic aorta unfolding process). Also, it has been shown that both aging and hypertension lead to unfolding of the aorta and the unfolding process with aging is accelerated in the presence of hypertension^20^. The current findings, therefore, may shed light on the role of aging in the increased prevalence of stroke in older patients with AF.

The present study has several limitations. Firstly, the number of patient-specific aorta models considered is small. There could be numerous age-, gender, or disease-dependent anatomic variations in human thoracic aortas^19–23^. Therefore, it is imperative that the current findings are confirmed for larger population of patient-specific aortic models. Despite the inherently probabilistic features of stroke incidence due to the complex behavior of a clot in blood stream, however, the variations in stroke propensity with aortic morphology is likely to be consistent over a large number of patients since it is based on deterministic aspects of hemodynamics or flow-structure relations.

Secondly, the curvature angle determination method adopted is simplified and only reflects the global pattern of the aortic configuration; i.e., it does not capture local variations in curvature. Hence, detailed image analysis approaches^19, 21, 24^ are necessary to more precisely correlate the aortic morphology with clot trajectory and stroke propensity. This also makes it more plausible to examine how altered cardiac hemodynamics is related to the flow field including pressure distribution in each aortic branch relative to aorta. Despite the lack of details in local curvature of the aorta morphology, however, the curvature angle defined here is indicative of the overall curvature of the aorta morphology such that increased pressure drop with increasing curvature angle was clearly demonstrated (Fig. 6).

Thirdly, although it was shown that the curvature angle of aorta can be an important determinant of AF-induced stroke propensity, it is necessary to investigate other possible risk factors of stroke propensity (e.g., aorta size, branching pattern, etc.) and their relative contributions. In fact, the aorta diameter considered in the present study was shown to be fairly constant (CV = SD/Mean = 9.5%). The effect of aorta size on stroke propensity will be an essential subject for the further study since aortic hemodynamics can be considerably influenced by the aorta size.

Fourthly, stroke is known to increase in frequency in older patients^1^ whose aortic stiffness tends to be higher. This justified our current approach that aortic wall was assumed to be rigid. While aging has been identified as an independent risk factor for AF development, AF is not just an elderly problem but also affects the younger generation^25^. Therefore, it is a logical next step to explore the role of aortic wall compliance in embolic stroke incidence through fluid-structure interaction analysis.

Lastly, although the AF hemodynamic profile considered are representatives of AF (i.e., reduced cardiac output and cycle length), AF is also characterized by irregular heartbeats^13^ which may lead to a variety of cardiac hemodynamic profiles. Furthermore, it has been reported that emboli to the brain can originate from various sources or cardiogenic embolism (cardiac chambers), arteriogenic embolism (proximal cerebral arteries or aortic arch), and paradoxical embolism (veins)^26^ which may affect pattern of clot dislodgment and hence clot motion in blood flow. Thus, further studies are necessary to understand the full role of hemodynamics in embolic stroke which could enable identification of patient-dependent, stroke-prone conditions.

Despite the limitations of the present study, it was clearly shown that AF-induced hemodynamic perturbation may differently affect clot trajectory and hence embolic propensity in LCCA. Although it is premature to directly translate the present study to physiology and clinical intervention, the small cohort of patient–specific computational models are hypothesis generating as to the potential role of aortic curvature which has not been previously proposed.

## Methods

### Image-based Aorta Models

The research was conducted according to the principles of the Declaration of Helsinki. The 3D aortic arch models (ascending aorta size is 24.3 ± 2.3 mm in diameter, Mean ± SD) for CFD simulations were reconstructed from patient computed tomography (CT) data by image processing algorithm including thresholding and region growing adopted in previous study^27^. The vascular surface was smoothed and simplified by MeshLab (an open source 3D mesh processing software) to remove noise. The tetrahedral computational mesh was constructed using an ANSYS meshing tool such that mesh density was commensurate with previous studies^17, 28^. This was done to ensure mesh-independent solutions that capture complex flow patterns in aortic arch configuration especially near curved and branching regions.

### Flow Modeling

Computational schemes and modeling methodologies have been described in detail in our previous studies^17, 28^. Briefly, the flow field was obtained from solving the Navier-Stokes Equations for incompressible Newtonian viscous fluid or blood with density and viscosity of 1060 kg/m^3^ and 0.0035 kg/m-s, respectively. It was previously verified that the effect of non-Newtonian viscosity in aortic turbulent flow model on changes in flow patterns was not discernable^29^. Various computational approaches including turbulence models have been proposed to simulate the aortic blood flow behavior^29–33^. The impact of turbulence modeling on the trajectory of a blood clot in the aortic arch has been discussed previously^17^. For the present simulations, the k-ω SST (shear stress transport) model was adopted to capture the complex turbulent behavior in the aortic arch.

The velocity profiles specified at the aortic inlet and boundary conditions at the outlet of each aortic branch and descending aorta were identical to the conditions adopted in a previous studies^17, 28^. Briefly, a velocity profile representing a normal cardiac hemodynamic condition was modeled and then the velocity profile was modified to represent an AF-induced hemodynamic perturbation that is commensurate with 30% and 40% reduced cardiac output and cycle length based on patient measurements^13^. A constant fraction of the inlet flow rate was imposed at each outlet based on measured flow rates^30^. The vessel wall was assumed to be rigid.

FLUENT 14.5 (a commercial CFD simulation software package from ANSYS, Inc.) was used to solve the governing equations. All simulations were performed on a Dell Workstation T7500 with two Intel Xeon processors and 24 GBytes of memory.

### Embolus Motion

The motion of a clot in blood flow was modeled based on the coupled Euler-Lagrange approach for a particle (dispersed phase) in the fluid flow (continuous phase) which is consistent with previous studies^17, 28, 31, 34^. Basically, trajectory of a clot was determined such that acceleration of the clot is balanced with the forces exerted on the clot which include drag force (i.e., resistance force caused by the relative motion of the clot through the surrounding blood), buoyancy force (i.e., upward force on the clot immersed in the blood), virtual mass force (i.e., force due to the difference in acceleration between dispersed phase or clot and continuous phase or blood), and force due to the pressure gradient in the flow field.

Theoretically, two-way coupling approach or momentum exchange between two phases (i.e., clot and blood) is more accurate but requires tremendously added computational cost since simultaneous simulations of numerous clot ejection scenarios may be inevitable from the clinical view point but are not plausible in the two-way coupling methodology^34^. Based on previous studies^17, 28, 34^, the interactions between clot and blood flow was simplified as one-way coupling. Interactions between embolus and vessel wall are also complicated biochemically and physically. Elastic collision was assumed which is consistent with previous studies^17, 28, 31^. Clot modeling methods were based on our previous studies where physical parameters and ejection scenarios of emboli are described in detail^17, 28^. Also, the stroke propensity was calculated by counting the number of clots transported into the LCCA for each patient and the statistical analysis (one-way ANOVA) was performed to evaluate the significance of the stroke propensity for the five patients.

### Curvature of Aorta Arch

It has been demonstrated that vascular curvature and swirling helical flow may play a substantial role in embolus transport within the carotid artery^34^. Thus, adequate measure of curve and torsion of aortic configurations is an essential element to systematically investigate the role of aorta morphology in clot trajectory and stroke propensity. Various methods have been proposed to measure curvature in vasculature^19–24, 35^. Here, we adopted a simple approach for the measure of aortic arch curve angle (Fig. 1C)^35^. In addition, torsion angle was also introduced (Fig. 1D) to account for 3D curvature of the aorta. As a simple overall indicator of the aortic curve and torsion, a curvature angle or sum of curve and torsion angles was defined such that higher curvature angles represent more severe tortuous patterns of aorta geometrical configurations.
